# Analyzing trends and future projections in fodder oats (*Avena sativa* L.) for quality seed production in India

**DOI:** 10.3389/fpls.2025.1525422

**Published:** 2025-04-07

**Authors:** Subhash Chand, Sanjay Kumar, Ajoy Kumar Roy, Dunna Vijay, Bishwa Bhaskar Choudhary, Indu, Pradeep Kumar, Rajiv Kumar Agrawal, Vijay Kumar Yadav, Pankaj Kaushal, Devendra Kumar Yadava, Ram Vinod Kumar, Awnindra Kumar Singh, Shahid Ahmed, Devendra Ram Malaviya, Rajesh Kumar Singhal, Birendra Prasad, Rahul Kapoor, Amit Kumar Jha, Ravish Panchta

**Affiliations:** ^1^ ICAR–Indian Grassland and Fodder Research Institute, Jhansi, India; ^2^ ICAR–National Research Centre on Seed Spices, Ajmer, Rajasthan, India; ^3^ ICAR–Indian Agricultural Research Institute, New Delhi, India; ^4^ G.B. Pant University of Agriculture and Technology, Pantnagar, India; ^5^ Punjab Agricultural University, Ludhiana, India; ^6^ Jawaharlal Nehru Krishi Vishwa Vidyalaya, Jabalpur, India; ^7^ Chaudhary Charan Singh Haryana Agricultural University, Hisar, India

**Keywords:** fodder oats, quality seed, varietal replacement rate, breeder seed, parental homogeneity

## Abstract

Oats (*Avena sativa* L.) is a multipurpose, popular, nutritionally rich cereal crop widely used for food, feed, and fodder. In India, it is cultivated on nearly 0.25 M ha in the northern, northwestern, and central regions and has recently expanded to the eastern region, mainly for fodder purposes. Breeder seed (BS) production data were collected from the AICRP on Forage Crops and Utilization (FC&U) for a period of 24 years (1998–1999 to 2021–2022). Several fodder oat varieties have been developed and introduced into the seed chain in India over the past 24 years to suit different agro-climatic conditions. However, analysis reveals a narrow genetic base at the varietal level, with a few old and popular varieties (Kent, OS-6, and OS-7) sharing > 70% of the genome in varietal development. To encourage the cultivation of new varieties and replace older ones, adequate BS production is vital to ensure a regular supply of quality seeds for sustainable livestock production, providing nutritious and cost-effective fodder. With a few exceptions, the amount of BS indent and the number of varieties has increased, indicating growing demand and awareness of new varieties. At the institutional level, Indian Grassland and Fodder Research Institute (IGFRI) (Jhansi) contributed the highest to BS production (29.8%), followed by Punjab Agricultural University (PAU) (Ludhiana; 13.7%), AAU (Anand; 10.8%), and G.B. Pant University of Agriculture and Technology (GBPUAT) (Pantnagar; 9.9%). A moderate varietal replacement rate (22.9%) was observed for recently developed varieties (< 5 years) over the past 3 years (2019–2020 to 2021–2022). However, their contribution has significantly increased from 0.2% (2018–2019) to 26.2% (2021–2022). We estimated certified seed production (194,040 q) for 2023–2024 based on the available BS (485.1 q), assuming the seed chain operates at 100% efficiency. This production could cover 0.19 M ha of fodder oats in 2024–2025. The ARIMA model estimated that BS production and the number of varieties in the seed chain would reach 734.2 q and 28, respectively, by 2026–2027. Additionally, breeding approaches and improved management practices for enhanced seed production were discussed, and a roadmap was proposed to meet the demand for quality fodder oat seed in India.

## 1 Introduction

Oats (*Avena* species) are self-pollinated crops belonging to the family Poaceae. All hexaploid oats were reported to have originated from a common center in Southwestern Asia. The cultivated oat, i.e., *Avena sativa* L. (2*n* = 6*x* = 42), is a natural allopolyploid (AACCDD) that evolved through several interspecific hybridization and polyploidization cycles. The wild species *Avena sterilis* L. and *Avena fatua* L. share the same genome as *A. sativa* (Boczkowska et al., 2016; Sood et al., 2022). *Avena byzantine* was the most dominant species in the primeval era, whereas *A. sativa* is now the predominant and widely cultivated species (Coffman, 1977).

Oat ranks sixth in global cereal production, following wheat, maize, rice, barley, and sorghum (FAO STAT, 2019). As a fodder crop, it exhibits excellent growth habits, rapid recovery after cutting, and high-quality herbage. Oat serves multiple uses, including grain, forage and fodder, straw, hay, silage, and chaff, and is mainly cultivated in marginal and submarginal lands. Oat is a primary fodder crop for livestock, covering nearly 74% of its global utilization (Burgess et al., 1972). In India, fodder oats are cultivated over approximately 0.25 million hectares (M ha), with the maximum acreage in Uttar Pradesh (34%), followed by Punjab (20%), Bihar (16%), Haryana (9%), and Madhya Pradesh (6%). The green and dry fodder productivity and seed yield range from 50 to 60, 12 to 15, and 2–3 t/ha, respectively.

Different oat varieties are cultivated either as a single-cut (harvested at 50% flowering stage) or multicut (first cut at 40–45 days. Followed by two subsequent cuts at 30–35-day intervals) types, depending on their fodder yield and regeneration capacity. With growing awareness of the benefits of oat grains, dual-purpose varieties (suitable for fodder with a cut at 30–35 days after sowing and grain harvested at maturity) are also gaining popularity. Green fodder contains crude protein (10%–11%), neutral detergent fiber (55%–63%), acid detergent fiber (30%–32%), cellulose (22%–25%), and hemicellulose (17%–20%) on a dry matter basis (Das et al., 2015). Oat straw is highly palatable, digestible, and acceptable to livestock, providing sufficient energy with moderate protein levels essential for livestock maintenance and growth (Ranjan et al., 2024). Additionally, its green fodder comprises more soluble carbohydrates readily transformed into high-quality silage (Chauhan and Singh, 2019; Kebede et al., 2023).

In India, the ICAR–All India Coordinated Research Project on Forage Crops and Utilization (AICRP on FC&U) evaluates all fodder varieties for their value for cultivation and use (Chand et al., 2020). The Indian seed multiplication program comprises three distinct classes: breeder, foundation, and certified seeds. It implements robust measures to maintain the quality of seeds throughout the multiplication process. The AICRP on FC&U plays a pivotal role in supervising and coordinating the maintenance and production of nucleus and breeder seed, as well as managing their supply network. This, in turn, indirectly contributes to the production of the required quantities of foundation (FS) and certified (CS) seeds [Available online at: https://seednet.gov.in/material/Indianseedsector.htm (Accessed 21.03.2024)]. The timely availability of high-quality seeds can enhance yield by 15%–20%, and when coupled with effective management practices, this increase could reach up to 45% [Available online at: https://seednet.gov.in/material/Indianseedsector.htm (Accessed 21.03.2024)]. The Government of India revises the breeder seed sale price yearly before the sowing season [Available online at: https://seednet.gov.in/material/Indianseedsector.htm (Accessed 21.03.2024)].

Forage crop varieties, including fodder oats, are shy seeders as they are mainly bred for high green and dry biomass. Oat is an introduced crop in India, and both primary and secondary introductions with high fodder biomass have been used as parents in breeding programs to enhance biomass production, adaptability, and fodder values. This study analyzed trends in BS production, varietal diversity, varietal replacement rate (VRR), and forecasted future seed demand in India. Additionally, future strategies for developing more adaptive, widely accepted, and diverse oat varieties for Indian farmers were discussed.

## 2 Materials and methods

### 2.1 Data collection

The data pertaining to the indent, allocation, and production of diverse fodder oat varieties in the Indian seed supply chain were sourced from the AICRP on FC&U annual reports spanning a period of 24 years (1998–1999 to 2021–2022) (Anonymous, 1999, 2000, 2001, 2002, 2003, 2004, 2005, 2006, 2007, 2008, 2009, 2010, 2011, 2012, 2013, 2014, 2015, 2016, 2017, 2018, 2019, 2020, 2021, 2022).

### 2.2 Seed replacement rate

It indicates the total cropped area sown using quality-ensured labeled seeds instead of farm-saved seeds. High seed replacement rate (SRR) denotes an efficient seed industry, extension programs, and sound market channels (Singh and Agrawal, 2018). SRR was calculated using the following formula:


$$\text{SRR}=\frac{\text{A}}{B}\times 100$$

Where, *A* represents the actual quantity of quality seed (certified/TFL) sown in an area (*q*), and *B* denotes the actual quantity of seed required for the entire production area (*q*).

### 2.3 Varietal replacement rate

This indicates the rate at which older varieties are being replaced by recently released high-yielding varieties. Genetic gain in terms of farm productivity can be improved significantly by rapidly disseminating improved varieties in place of older ones (Veettil et al., 2018). The VRR for the previous 3 years (2019–2020 to 2021–2022) is calculated using the following formula:


$$\text{VRR}=\frac{\text{X}}{Y}\times 100$$

Where, *X* is BS indent (*q*) of a recently (< 5, < 15, and > 15 years) released varieties of a given crop for the calculated years.


Y=Total BS indent of all varieties of a given crop(q)for the calculated years

The contribution of older but popular varieties to BS indent, along with the trend of varieties categorized by age (< 5, 5–15, and > 15 years), was analyzed for the past 5 years (2017–2018 to 2021–2022) to assess the impact of recently notified varieties.

### 2.4 Seed multiplication ratio

SMR is the ratio of seed output to seed input in a quantified area. A higher SMR helps reduce the cost of seed production while increasing yield. The SMR was calculated using the following equation, as suggested by Van Gastel et al. (2002).


$$\text{P}=\frac{\text{X}}{Y}$$

Where *P* is the seed multiplication ratio, *X* is pure seed yield (kg), and *Y* is the seed rate (kg). An SMR of 1:20 was taken for the oat crop (Chauhan et al., 2017).

### 2.5 Foundation and certified seed production estimation

FS and CS were calculated using the following formulas, and 1:20 SMR was used to convert from BS to CS (Chauhan et al., 2017).


FS production (q)=Breeder seed quantity (q)×20 

CS production (q)=Foundation seed quantity (q)×20

### 2.6 Estimated area under quality seed

It is estimated based on the total cultivable area sown with available quality seeds, using a standard seed rate of 100 kg/ha for fodder cultivation (Chauhan et al., 2017).


$$\begin{array}{c} \text{Area coverage }(\text{ha})\\ =\text{Total quality seeds produced }(\text{kg})\;/\text{ Seed rate }(\text{kg}/\text{ha}) \end{array}$$

### 2.7 Forecasting the BS production and varieties

The forecasting model developed by Box and Jenkins (1970), known as the Autoregressive Integrated Moving Average (ARIMA) model, was used to predict short-term breeder seed production and the demand for indented varieties in the fodder oat seed chain. The ARIMA is an extrapolation method that relies on historical time series data of underlying variables. In general, the model can be expressed as follows.

Let *Y_t_* be a discrete-time series variable (breeder seed production and number of varieties released) that takes different values over some time. The corresponding ARIMA model for the *Y_t_* series, with an autoregressive order of *p* {AR(P)} and a moving average order of *q* {MA(Q)}, can be expressed as:


$$Y_{t}=\alpha_{0}+\alpha_{1}Y_{t-1}+\alpha_{2}Y_{t-2}+\ldots +\alpha_{p}Y_{t-p}+\epsilon_{t}+\beta_{1}\epsilon_{t-1}+\beta_{q}\epsilon_{t-q}+V_{t}$$

*Y_t_* is the response variable at time *t*, *Y_t_*_–1_, *Y_t_*_–2_……*Y_t_*_–_ *_p_* are the respective variables at different times with lags; $\;\alpha_{0}$ , $\;\alpha_{1}$ … $\;\alpha_{p},\;$ $\beta_{1}.\beta_{q}$ are the coefficients, and 𝜀𝑡 is the error factor of AR model, and $V_{t}$ is error term for MA model. To identify the order of *p* and *q*, the Autocorrelation Function (ACF) and Partial Autocorrelation Function (PACF) were used.

The order of an ARIMA model is usually denoted by the notation ARIMA (*p*, *d*, *q*), where *p* is the order of the autoregressive part, *d* is the order of the differencing, and *q* is the order of the moving-average process. The ARIMA model was formulated after transforming the variable under forecasting into a stationary series. The stationary series was the set of values that varied over time around a constant mean and constant variance. The Augmented Dickey–Fuller test for the first differenced series shows that the production and number of varieties series was integrated into order one. Based on the significant parameter with minimum values of the Akaike Information Criterion (AIC), the ARIMA (0, 1, 1) model was selected to forecast breeder seed production and a number of varieties (**Supplementary Figures S1A, B**).

### 2.8 Data analysis and interpretation

Microsoft Excel (2013 version) was used for data analysis, and various parameters were calculated and interpreted to assess BS indent and production, their status (surplus/deficit), and the notified varieties in the seed supply chain since 1998–1999. In addition, BS indent and production trends, along with the contribution of different centers to BS allocation and production, were estimated for fodder oats in India.

## 3 Results

### 3.1 Varietal diversity and adaptability

**Figure 1:**
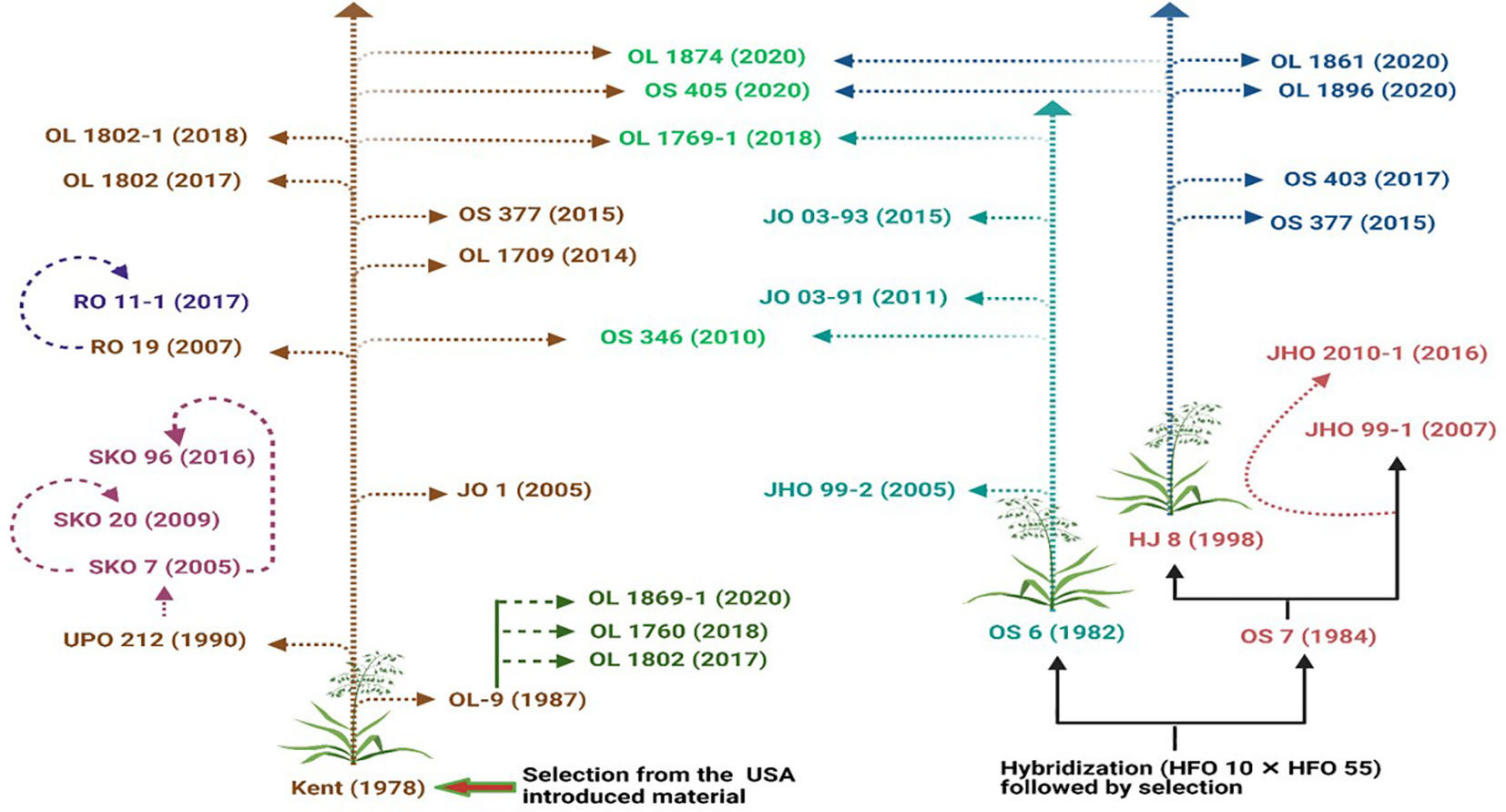
Continuous development of fodder oat varieties using the most popular parent varieties—Kent, OS-6, and OS-7. Kent contributes about 42% to varietal development programs in India, followed by OS-7 and OS-6. The values in parentheses indicate the varietal release/notification year, and the same color pattern represents the same source of origin.

Varietal diversification influences crop productivity and sustainability across different agroecological conditions by mitigating the adverse effects of biotic and abiotic stresses. In India, more than 60 fodder oat varieties were developed, released, and notified from 1976 to 2022, with 45 of these entering the seed supply chain (**Table 1**). Until 2010–2011, only a limited number of varieties (< 10) were in the seed supply chain; however, this number has grown significantly, reaching 23 varieties by 2019–2020 (**Figure 1**).

**Table 1:** Detailed description of indented fodder oat varieties in the seed supply chain in India from 1998–99 to 2021–22 (Data source: Roy et al., 2020).

S.N.	Variety	Year of release/notification	Breeding method/source	Parent institute	Area of adoption	Specific features (if any)
1.	HFO-114	1976	Selection from germplasm line	CCSHAU, Hisar	HR	Multicut type
2.	Kent	1978	Selection from the USA germplasm	PAU, Ludhiana	Pan India	Resistant to lodging and seed shattering, dual-type
3.	Palampur-1	1980	Pure-line selection from Algerian germplasm	CSKHPKV, Palampur	Lower and mid hills of HP	Resistant to powdery mildew, multicut type
4.	OS-6	1982	Pedigree method (HFO-10 × HFO-55)	CCSHAU, Hisar	Pan India	Resistant to major diseases and pests, single-cut type
5.	UPO-94	1982	Single-plant selection from ECOGP-73–M-94	GBPUAT, Pantnagar	PN, HR, RJ, Tarai region of UK, Western UP, MP, GJ, MH, and CG	Multicut, resistant to rust, smut, and leaf blight diseases, multicut type
6.	OS-7	1984	Pedigree method (HFO-10 × HFO-55)	CCSHAU, Hisar	Pan India	Tall and fast-growing, single-cut type
7.	OL-9	1987	Pedigree method (NP Hybrid × Kent)	PAU, Ludhiana	PN	Suitable for two cuts, dual type
8.	JHO-822	1989	Pedigree method (IGO-4268 × Indio)	IGFRI, Jhansi	UP, MP, GJ, and MH	Multicut type
9.	UPO-212	1990	Pedigree method (VS-1492 × Kent)	GBPUAT, Pantnagar	PN, HR, RJ, Tarai region of UK, Western UP, MP, GJ, MH, and CG	Tolerant to seed shattering, multicut type
10.	HJ-8	1998	Pedigree method ([OS-7 × S-3021 P-15])	CCSHAU, Hisar	HR	Fast growth and better regeneration, dual-type
11.	JHO-851	1998	Single-plant selection from the Japanese-introduced material	IGFRI, Jhansi	Pan India	High regeneration ability, multicut type
12.	SKO-7	2005	Pure-line selection from the UPO-212	SKUAST, Srinagar	Temperate and high-altitude regions of JK	Resistant to Downy mildew and Crown rust diseases and to aphids and Armyworm, dual type
13.	JHO-99-2	2005	Pedigree method (OS-6 × JHO-851-1)	IGFRI, Jhansi	PN, HR, RJ, BH, UK, OR, WB, Eastern UP, and AS	Quality fodder and digestibility, single-cut type
14.	JO-1	2005	Pedigree method (Kent × UPO-50)	JNKVV, Jabalpur	MP, part of UP, MH, GJ, and CG	Multicut type
15.	JHO-2000-4	2006	Interspecific hybridization followed by induced polyploidy and Pedigree method (Derivative of the cross JHO-851 (*A. sativa*) × *A. maroccana*-16/30B)	IGFRI, Jhansi	PN, RJ, HR, UK, JK, AP, KT, hilly tracts of TN, AS, WB, JH, OR, and BH	Wider adaptability, single-cut type
16.	JHO-99-1	2007	Pedigree method (OS-7 × IGO-3201139-19)	IGFRI, Jhansi	Hilly areas of JK, HP, UK, and the Nilgiris hills of TN	Resistant to grasshoppers and aphids, single-cut type
17.	RO-19	2007	Selection from oat variety Kent	MPKV, Rahuri	PN, HR, RJ, Tarai region of UK, Western UP, MP, GJ, MH, and CG	Resistant to leaf spot disease, multicut type
18.	SKO-20	2009	Pedigree method (EC-13178 × Sabzaar)	SKUAST, Srinagar	JK	Single-cut/multi/dual
19.	OS-346	2010	Pedigree method (OS-6 × Kent)	CCSHAU, Hisar	MP, GJ, and MH	Suitable for single-cut
20.	NDO-1	2011	Mass selection (Local collection from Kothi, Barabanki, U.P.)	ANDUAT, Ayodhya	UP	Highly palatable, single-cut type
21.	JO-03-91	2011	Pedigree method (OS-6 × JHO-822)	JNKVV, Jabalpur	MP	Single cut type
22.	NDO-2	2013	Pedigree method (JO-6 × OL-1389)	ANDUAT, Ayodhya	UP	Highly palatable, dual type
23.	OL-1709	2014	Pedigree method (Kent × OL-1245)	PAU, Ludhiana	PN	Tolerant to leaf blight and aphids
24.	OS-377	2015	Pedigree method (HJ-8 × Kent)	CCSHAU, Hisar	UP, MH, GJ, CG, and MP	Single-cut type
25.	JO-03-93	2015	Pedigree method (OS-6 × JHO-822)	JNKVV, Jabalpur	MP	Single-cut type
26.	JHO-2009-1	2016	Single-plant selection from EC-425113	IGFRI, Jhansi	UP, MP, MH, and GJ	Moderately resistant to *Sclerotium* rot, leaf blight, and nematodes, single-cut type
27.	JHO-2010-1	2016	Pedigree method (OS-7 × IGO-320)	IGFRI, Jhansi	AP, KT, and TN	Moderately resistant to *Sclerotium* rots, leaf blight, and powdery mildew, single-cut type
28.	SKO-96	2016	Pedigree method (Sabzaar × Banquo)	SKUAST, Kashmir	Hilly areas of JK, UK, and HP	High crude protein yield, single-cut type
29.	UPO-06-01	2016	Pedigree method ([UPO-201/211//201]-56-1-15)	GBPUAT, Pantnagar	Northwest plains and lower hills of Uttarakhand	Resistance to major diseases and pests, single-cut type
30.	RO-11-1	2017	Pure-line selection from RO-19	MPKV, Rahuri	WB, OR, JH, BH, UP, MN, AS, RJ, PN, HR, UK, MH, MP, GJ, CG, TL, AP, TN, and KT	High response to nitrogen fertilizer, single-cut type
31.	OL-1802	2017	Pedigree method (OL-9 × Kent)	PAU, Ludhiana	PN, HR, RJ, plains of UP, and UK	Suitable for single-cut system
32.	OS-403	2017	Pedigree method (HJ-8 × Algerian)	CCSHAU, Hisar	AS, MN, OS, WB, Eastern UP, BH, JH, TL, AP, KT, and TN	Enhanced nutritional quality, single-cut type
33.	OL-1760	2018	Pedigree method (OL-9 × OL-125)	PAU, Ludhiana	TL, AP, TN, and KT	Suitable for single-cut system
34.	OL-1802-1	2018	Pedigree method (Kent × OL-125)	PAU, Ludhiana	PN, HR, RJ, plains of UP, and UK	Suitable for single-cut system
35.	OL-1769-1	2018	Pedigree method (Kent × OS-6)	PAU, Ludhiana	UP, MH, MP, GJ, and CG	Suitable for single-cut system
36.	JHO-2012-2	2018	Individual plant selection from EC-498707	IGFRI, Jhansi	AP, KT, and TN	Moderately resistant to leaf blight, single-cut type
37.	JO-04-315	2018	Mutation breeding (irradiation of entry JO-1 with 250 Gy)	JNKVV, Jabalpur	MP and Bundelkhand region of UP	Resistant to lodging and nonshattering type, single-cut type
38.	JHO-2015-1	2019	Pedigree method (UPO-90 × IG-02-70)	IGFRI, Jhansi	Hilly areas of JK, HP, and UK	Resistant to lodging and nonshattering type, single-cut type
39.	JO-5	2019	Mutation breeding	JNKVV, Jabalpur	MP	Multicut type
40.	OL-1869-1	2020	Pedigree method (OL-9 × OL-125)	PAU, Ludhiana	PN, HR, RJ, Tarai region of UK, Western UP, MP, GJ, MH, and CG	Single-cut type
41.	OL-1861	2020	Pedigree method (HJ-8 × OL-1610)	PAU, Ludhiana	PN, HR, RJ, AS, MN, BH, JH, OR, UP, MP, GJ, MH, TN, KT, and AP	Single-cut type
42.	OS-405	2020	Pedigree method (HJ-8 × Kent)	CCSHAU, Hisar	Hilly areas of JK, HP, and the UK	Timely sown, single-cut type
43.	OL-1876-2	2020	Pedigree method (JHO-2001 × EC-209616)	PAU, Ludhiana	AS, OS, JH, and eastern UP	Dual type, high tillering ability, moderately resistant to leaf blight
44.	OL-1896	2020	Pedigree method (HJ-8 × OL-1610)	PAU, Ludhiana	RJ, HR, PN, and Tarai part of UK	Moderately resistant to leaf blight, single-cut type
45.	OL-1874	2020	Pedigree method (HJ-8 × Kent)	PAU, Ludhiana	RJ, HR, PN, and Tarai part of UK	Moderately resistant to leaf blight, multicut

*SKO-7*, Sabzar; *RO-19*, Phule Haritha; *SKO-20*, Shalimar Fodder Oat-1; *OL-1709*, OL-10; *SKO-96*, Shalimar Fodder Oat-3; *RO-11-1*, Phule Surabhi; *OL-1760*, OL-11; *OL-1802-1*, OL-12; *JHO-2012-2*, BJ-2012-2; *OL-1869-1*, OL-13; *OL-1874*, OL-14; *PN*, Punjab; *RJ*, Rajasthan; *HR*, Haryana; *UK*, Uttarakhand; *BR*, Bihar; *CG*, Chhattisgarh; *MP*, Madhya Pradesh; *UP*, Uttar Pradesh; *JH*, Jharkhand; *WB*, West Bengal; *HP*, Himachal Pradesh; *AS*, Assam; *GJ*, Gujarat; *MH*, Maharashtra; *TN*, Tamil Nadu; *KT*, Karnataka; *AP*, Andhra Pradesh; *JK*, Jammu and Kashmir; *OR*, Orissa; *TL*, Telangana.

During the 1970s and 1980s, the selection method was used to develop oat varieties from exotic germplasm. For instance, the variety HFO-114 (1976) was selected from exotic germplasm and recommended for cultivation in Haryana state. The most popular fodder oat variety “Kent” (1978) was developed through the selection of introduced material from the USA (**Table 1**). With the expansion of breeding programs for fodder oats in other institutions, 11 varieties had been notified by the year 2000. Similarly, 34 improved fodder oat varieties were released for different agroecological conditions and entered into the seed chain from 2000 to 2020, with 21 of these varieties developed and released in the last 5 years (2015 to 2020) (**Table 1**). As a winter fodder crop, oat is traditionally cultivated in the north-western and central Indian states (Haryana, Punjab, Rajasthan, Uttar Pradesh, Tarai region of Uttarakhand, Maharashtra, Gujarat, Chhattisgarh, and Madhya Pradesh) covering more than 60% of the total cultivable area under fodder oat with maximum fodder productivity. However, certain varieties (JHO-99-2, RO-11-1, OS-403, and JHO-2000-4) were specifically developed and released for the eastern and northeastern Indian states (Bihar, Odisha, West Bengal, Assam, Manipur and Jharkhand). In contrast, varieties such as JHO-2010-1, JHO 2000-4, RO-11-1, OL-1861, JHO-2012-2, OL-1760, and OL-403 were released for the southern Indian states, including Andhra Pradesh, Telangana, Karnataka, and Tamil Nadu (**Table 1**).

**Figure 2:**
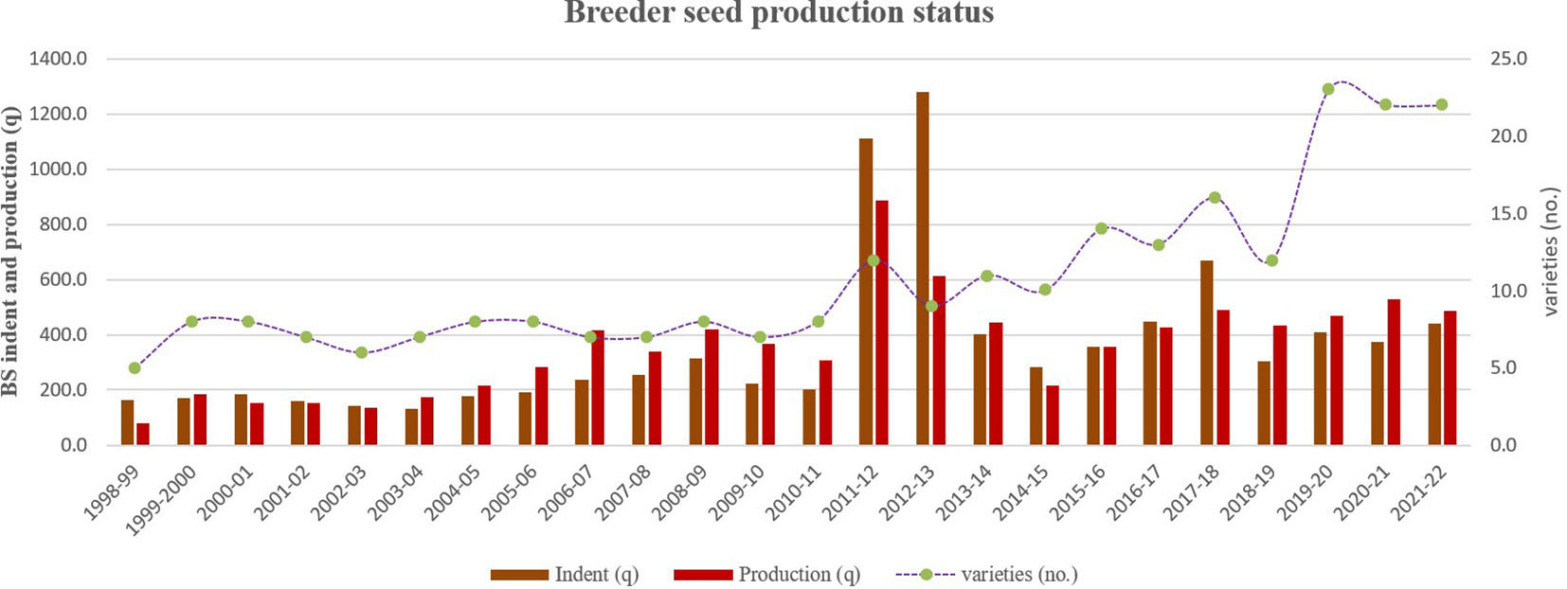
Fodder oat BS indent (*q*), production (*q*), and the number of varieties in the seed chain in India from 1998–1999 to 2021–2022.

Hybridization, followed by selection, is the most common breeding method used in self-pollinated crops. Kent, HJ-8, and OS-6 varieties were predominantly used as parents, contributing to more than 70% of the total released varieties (**Figure 2**). The pedigree method was frequently employed for varietal development, resulting in the development of > 35 varieties by 2022. Green fodder productivity is influenced by agro-climatic conditions, agronomic practices, and soil types. However, HJ-8 (600–680 q/ha), exhibited the highest green fodder production, followed by UPO-212, RO-19, and JHO-2009-1 (all ranging from 550 to 600 q/ha). Fodder oat varieties are developed for three categories: single-cut, multicut, and dual-type. The majority of developed varieties fall under the single-cut category, followed by multicut and dual-type varieties. For example, HFO-114, Palampur-1, UPO-94, JHO-822, UPO-212, JHO-851, JO-1, RO-19, OL-1874, and JO-5 were developed as multicut type varieties, whereas Kent, OL-9, HJ-8, SKO-7, SKO-20, NDO-2, and OL-1876-2 were bred for dual-purpose use (**Table 1**).

### 3.2 BS production vs. indent

The number of oat varieties in the seed chain remained below 10 from 1998–1999 to 2010–2011. However, it increased to over 20 in the past 3 years (2019–2020 to 2021–2022) due to the concerted efforts of ICAR Institutes and SAUs in developing agro-climatic zone-specific varieties (**Figure 2**). Initially, only six varieties developed during 1981–1990 were part of the seed chain, but this number gradually rose to eight during 2001–2010 and to 26 during 2011–2020. Most of these varieties were developed by PAU (Ludhiana), Chaudhary Charan Singh Haryana Agricultural University (CCS HAU) (Hisar), IGFRI (Jhansi), Jawaharlal Nehru Krishi Vishwa Vidyalaya (JNKVV) (Jabalpur), and Mahatma Phule Krishi Vidyapeeth (MPKV) (Rahuri) (**Table 1**).

The 24 years of BS indent and production data were grouped into six phases, each spanning 4 years. The first phase (1998–1999 to 2001–2002) was used as the base year block to determine the percent change in BS indent and production in subsequent phases (**Table 2**). Fodder oat BS indent and production increased significantly over the years, except for phase II (2002–2003 to 2005–2006), where the indent decreased by − 4.9% compared to the base year. BS production was surplus against the indent in all phases except the first (1998–1999 to 2001–2002), fourth (2010–2011 to 2013–2014), and fifth (2014–2015 to 2017–2018) phases, where production decreased by − 15.8%, − 24.9%, and − 15.2%, respectively. Overall, total fodder oat BS production (8,628.0 q) was almost equal to the indent (8,569.6 q) over the past 24 years.

**Table 2:** Fodder oat BS indent, production and percent change at 4-year intervals in India from 1998–1999 to 2021–2022.

Years^a^	Indent		Production		Net (production-indent)	
	Quantity (*q*)	% change	Quantity (*q*)	% change	Surplus/deficit (*q*)	Surplus/deficit (%)
Phase I (1998–1999 to 2001–2002)	677.0	–	570.2	–	− 106.7	− 15.76%
Phase II (2002–2003 to 2005–2006)	643.6	− 4.9	806.8	+ 41.5	+ 163.2	+ 25.36%
Phase III (2006–2007 to 2009–2010)	1,030.8	+ 52.3	1,540.1	+ 170.1	+ 509.4	+ 49.42%
Phase IV (2010–2011 to 2013–2014)	2,995.1	+ 342.4	2,248.3	+ 294.3	− 746.8	− 24.93%
Phase V (2014–2015 to 2017–2018)	1,756.8	+ 159.5	1,489.8	+ 161.3	− 267.0	− 15.20%
Phase VI (2018–2019 to 2021–2022)	1,524.9	+ 125.3	1,914.4	+ 235.7	+ 389.6	+ 25.62%
**Total**	**8,628.0**		**8,569.6**		**− 58.4**	**− 0.68%**

a Phase I was considered the base year, and the percent change was calculated relative to this base year block.

Fodder oat BS production consistently exceeded the indent since 1998–1999, except for six unusual years—1998–1999, 2000–2001, 2011–2012, 2012–2013, 2014–2015, and 2017–2018—when production was significantly lower by − 51.5%, − 17.2%, − 20.3%, − 52.2%, − 23.5%, and − 26.6%, respectively, compared to the indent received. This shortfall was probably due to an abrupt increase in BS indent and the unavailability of sufficient nucleus seed followed by adverse weather conditions (**Figure 2**). However, production was marginally lower than indent in 4 years: 2001–2002 (− 3.6%), 2002–2003 (− 6.0%), 2015–2016 (− 0.6%), and 2016–2017 (− 4.6%). Over the past 24 years, BS indent and production have gradually increased, starting from 163.6 to 79.4 q in 1998–1999 and reaching up to 438.9 to 485.1 q in 2021–2022, respectively. However, exceptionally high indents and productions were recorded during 2011–2012 (1,112.7 and 887.3 q) and 2012–2013 (1,278.1 and 611.3 q). This surge was attributed to a higher indent for specific varieties (Kent, UPO-212, JHO-851, and JHO-99-2) from the indenters (NDDB, DADH, IFFED, KVSSL, NSC, NSAI, and other milk cooperatives through the state department of agriculture) to the Department of Agriculture, Cooperation and Farmers Welfare, Govt. of India (DAC).

### 3.3 Varietal BS indent vs. production dynamics

**Figure 3:**
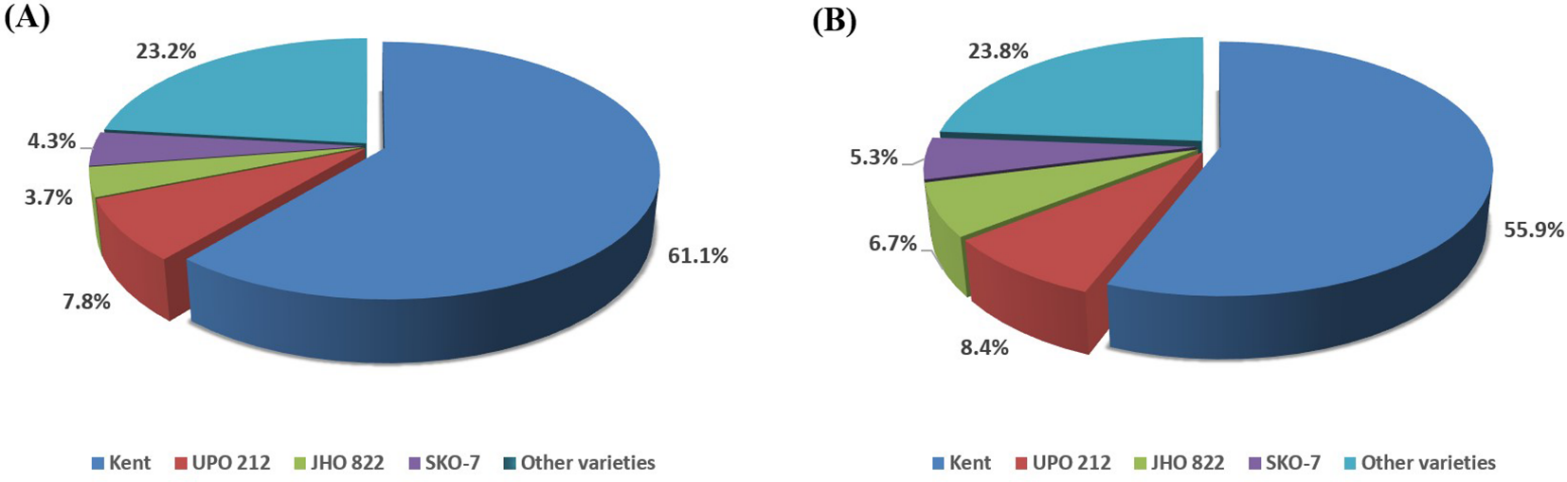
Contribution of the four leading fodder oat varieties (Kent, UPO 212, JHO 822, and SKO 7) to BS demand **(A)** and production **(B)** in India from 1998–1999 to 2021–2022.

**Figure 4:**
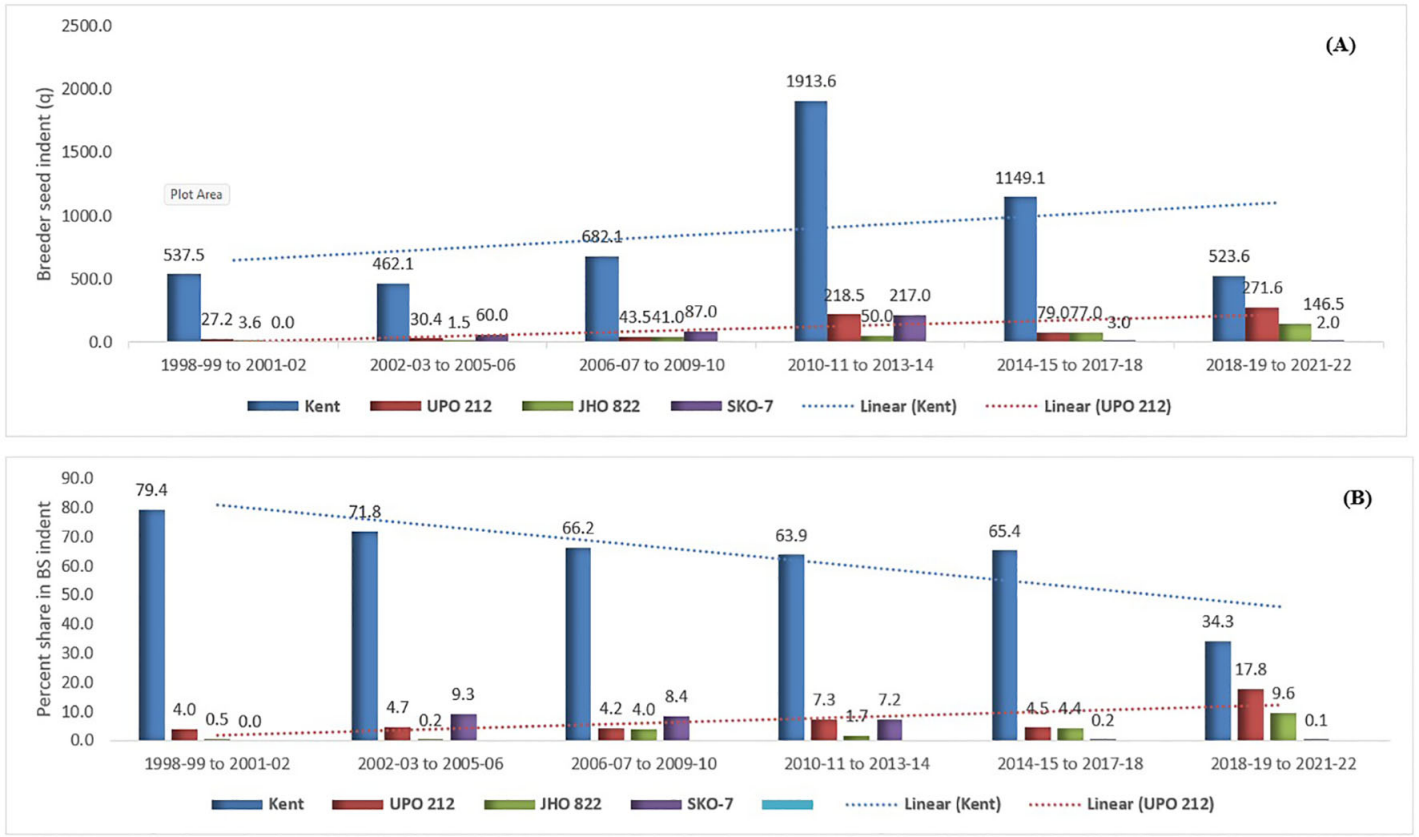
Breeder seed indent of the four foremost fodder oat varieties **(A)** and their percentage contribution **(B)** to the total BS indent in India from 1998–1999 to 2021–2022.

BS is produced annually by institutes whose varieties are indented by various seed multiplication agencies. Over the years, the number of varieties in the seed chain has increased; however, older varieties (Kent, UPO-212, JHO-822, OS-6, JHO-851, and HJ-8), notified before 2000, continue to contribute considerably to BS indent and production. In the first phase, Kent had the highest BS indent and production (79.4% and 78.8%), followed by OS-6 (7.0% and 6.2%) and HJ-8 (3.0% and 3.6%) (**Table 3**). Kent has remained the leading mega variety across all six phases, contributing more than 60% of the total BS indent and production until the fifth phase. The highest BS production contributions were recorded for Kent (62.0%), followed by SKO-7 (17.0%) in the second phase; Kent (64.3%) and JHO-822 (8.4%) in the third; Kent (59.7%) and JHO-99-2 (7.4%) in the fourth; Kent (60.2%) and JHO-822 (6.0%) in the fifth; and Kent (31.9%) and UPO-212 (15.9%) in the sixth phase (**Table 3**). Over the last 24 years, four oat varieties—Kent, UPO-212, JHO-822, and SKO-7—have accounted for over 75% of the combined BS indent and production (**Figures 3A, B**). Among the varieties, Kent had the highest contribution to BS indent and production (61.1% and 55.9%), followed by UPO-212, JHO-822, and SKO-7, which together accounted for 15.8% and 20.4%. However, Kent’s percentage contribution to the total BS indent has gradually declined over the years (**Figure 4**).

**Table 3:** Varietal BS indent and production of fodder oat varieties, along with their systematic phase-wise contribution in India from 1998–1999 to 2021–2022.

Variety	Year of release	Phase I (1998–1999 to 2001–2002)			Phase II (2002–2003 to 2005–2006)			Phase III (2006–2007 to 2009–2010)			Phase IV (2010–2011 to 2013–2014)			Phase V (2014–2015 to 2017–2018)			Phase VI (2018–2019 to 2021–2022)			Overall		
		*I*	*P*	Net^a^	*I*	*P*	Net^a^	*I*	*P*	Net^a^	*I*	*P*	Net^a^	*I*	*P*	Net^a^	*I*	*P*	Net^a^	*I*	*P*	Net^a^
HFO-114	1976	13.2 (1.9)	8.2 (1.4)	− 5.0	11.0 (1.7)	9.7 (1.2)	− 1.3													24.2 (0.3)	18.0 (0.2)	− 6.2
Kent	1978	537.4 (79.4)	449.5 (78.8)	− 87.9	462.1 (71.8)	499.1 (61.8)	37.0	682.1 (66.2)	990.2 (64.3)	308.1	1,913.6 (63.9)	1,343.0 (59.7)	− 570.6	1,149.1 (65.4)	897.5 (60.2)	− 251.6	523.6 (34.3)	610.3 (31.9)	86.7	5,267.8 (61.1)	4,789.7 (55.9)	− 478.1
Palampur-1	1980																2.00 (0.13)	2.40 (0.13)	0.4	2.00 (0.02)	2.40 (0.02)	0.4
OS-6	1982	47.2 (7.0)	35.2 (6.2)	− 12.0	28.8 (4.5)	28.9 (3.6)	− 0.1	76.2 (7.4)	64.1 (4.2)	− 12.1	44.0 (1.5)	40.7 (1.8)	− 3.3	40.0 (2.3)	42.3 (2.8)	2.3	5.0 (0.3)	10.2 (0.5)	5.2	241.2 (2.8)	221.5 (2.6)	− 19.7
UPO-94	1982				5.6 (0.9)	9.0 (1.1)	3.4		15.0 (1.0)	15.0										5.6 (0.06)	24.0 (0.3)	18.4
OS-7	1984	12.8 (1.9)	13.2 (2.3)	0.4	3.2 (0.5)															16.0 (0.2)	13.2 (0.2)	− 2.8
OL-9	1987													2.2 (0.1)	3.0 (0.2)	0.8	3.0 (0.2)	3.0 (0.2)		5.2 (0.06)	6.0 (0.07)	0.8
JHO-822	1989	3.6 (0.5)	12.0 (2.1)	8.4	1.5 (0.2)	27.2 (3.3)	25.7	41.0 (3.0)	130.0 (8.4)	89.0	50.0 (1.8)	61.9 (2.7)	11.9	77.0 (4.4)	89.9 (6.0)	12.9	146.5 (9.6)	249.5 (13.0)	103	319.6 (3.7)	570.6 (6.6)	251.0
UPO-212	1990	27.2 (4.0)	17.0 (3.0)	− 10.2	30.4 (4.7)	53.0 (6.6)	22.6	43.5 (4.2)	105.0 (6.8)	61.5	218.5 (7.3)	162.0 (7.2)	− 56.5	79.0 (4.5)	79.0 (5.3)		271.6 (17.8)	305.0 (15.9)	33.4	670.2 (7.8)	721.0 (8.4)	50.8
JHO-851	1998	15.0 (2.2)	15.0 (2.6)	0	15.0 (2.3)	19.1 (2.4)	4.1	30.0 (2.9)	62.0 (4.0)	32.0	171.0 (5.7)	108.5 (4.8)	− 62.5	16.0 (0.9)	33.7 (2.2)	17.7		4.1 (0.2)	4.1	247.0 (2.9)	242.4 (2.8)	− 4.6
HJ-8	1998	20.5 (3.0)	20.3 (3.6)	− 0.17	26.0 (4.0)	25.2 (3.1)	− 0.8	51.0 (4.9)	52.8 (3.4)	1.8	49.0 (1.6)	51.4 (2.3)	2.4	27.6 (1.6)	28.6 (1.9)	1.0	16.8 (1.1)	32.5 (1.7)	15.7	190.9 (2.2)	210.8 (3.0)	19.9
JHO-99-2	2005							5.0 (0.5)	8.0 (0.5)	3.0	187.0 (6.2)	165.6 (7.3)	− 21.4	41.8 (2.4)	43.7 (2.9)	1.9	16.5 (1.1)	9.0 (0.5)	− 7.5	250.3 (2.9)	226.3 (2.6)	− 24
JO-01	2005								20.0 (1.3)	20.0					6.5 (0.4)	6.5		19.5 (1.0)	19.51		46.0 (0.5)	46.0
SKO-7	2005				60.0 (9.3)	135.5 (16.8)	75.5	87.0 (8.4)	93.0 (6.0)	6.0	217.0 (7.2)	215.0 (9.6)	− 2.0	3.0 (0.2)	5.0 (0.3)	2.0	2.0 (0.1)	2.0 (0.1)		369.0 (4.3)	450.5 (5.3)	81.5
JHO-2000-4	2006							15.0 (1.4)		− 15.0	15.0 (0.5)	10.9 (0.5)	− 4.1	62.5 (3.6)	31.5 (2.1)	− 31.0	10.0 (0.7)	21.0 (1.1)	11	102.5 (1.2)	63.4 (0.7)	− 39.0
JHO-99-1	2007										10.0	7.5 (0.3)	− 2.5	6.5 (0.3)	8.0 (0.5)	1.5	5.0 (0.3)	9.0 (0.5)	4	21.5 (0.2)	24.5 (0.3)	3.0
RO-19	2007										66.0 (2.2)	33.6 (1.5)	− 32.4	40.0 (2.3)	23.6 (1.6)	− 16.4	10.1 (0.6)	52.7 (2.7)	42.5	116.1 (1.3)	109.9 (1.3)	− 6.2
SKO-20	2009										11.0 (0.4)	21.0 (0.9)	10.0	30.0 (1.7)	42.0 (2.8)	12.0	105.5 (6.9)	106.5 (5.6)	1	146.5 (1.7)	169.5 (2.0)	23
OS-346	2010										18.0 (0.6)	20.0 (0.9)	2.0				2.5 (0.2)	15.5 (0.8)	13	20.5 (0.2)	35.5 (0.4)	15
JO-03-91	2011										18.0 (0.6)		− 18					5.0 (0.3)	5	18.0 (0.2)	5.0 (0.05)	− 13
NDO-1	2011										7.0 (0.2)	6.7 (0.3)	− 0.3	9.0 (0.5)	0.4 (0.03)	− 8.5				16.0 (0.2)	7.2 (0.08)	− 8.8
NDO-2	2013													5.0 (0.3)		− 5.0	1.0 (0.1)	0.5 (0.03)	− 0.5	6.0 (0.1)	0.50 (0.0)	− 5.5
OL-1709	2014													43.0 (2.4)	64.0 (4.3)	21.0	60.5 (4.0)	67.5 (3.5)	7	103.5 (1.2)	131.5 (1.5)	28
JO-03-93	2015													25.0 (1.4)	5.0 (0.3)	− 19.9	2.5 (0.2)	5.00 (0.3)	2.5	27.5 (0.3)	10.0 (0.1)	− 17.5
OS-377	2015													25.0 (1.4)	29.2 (2.0)	4.2	16.2 (1.1)	41.7 (2.9)	25.5	41.2 (0.5)	70.9 (0.8)	29.7
JHO-2009-01	2016													25.0 (1.4)	5.6 (0.4)	− 19.4	31.0 (2.0)	57.7 (3.0)	26.7	56.0 (0.6)	63.3 (0.7)	7.3
JHO-2010-01	2016													20.0 (1.1)	5.4 (0.4)	− 14.6		18.0 (0.9)	18	20.0 (0.2)	23.4 (0.3)	3.4
SKO-96	2016													30.0 (1.7)	34.00 (2.9)	4.0				30.0 (0.3)	34.0 (0.4)	4
UPO-06-1	2016																13.3 (0.9)		− 13.3	13.3 (0.1)		− 13.3
OS-403	2017																84.9 (5.5)	70.5 (3.7)	− 14.4	84.9 (0.1)	70.5 (0.8)	− 14.4
OL-1802	2017																20.5 (1.3)	9.0 (0.5)	− 11.5	20.5 (0.2)	9.0 (0.1)	− 11.5
RO-11-1	2017														11.7 (0.8)	11.7	27.0 (1.8)	20.2 (1.0)	− 6.8	27.0 (0.3)	31.9 (0.4)	4.9
JHO-2012-2	2018																2.0 (0.1)	5.2 (0.3)	3.2	2.0 (0.02)	5.2 (0.06)	3.2
JO-04-315	2018																4.5 (0.3)	1.7 (0.1)	− 2.8	4.5 (0.05)	1.7 (0.02)	− 2.8
OL-1760	2018																9.0 (0.6)	20.5 (1.1)	11.5	9.0 (0.10)	20.5 (0.2)	11.5
OL-1802-1	2018																39.5 (2.6)	38.5 (2.0)	− 1.0	39.5 (0.5)	38.5 (0.4)	− 1
OL-1769-1	2018																24.0 (1.6)	34.0 (1.8)	10	24.0 (0.3)	34.0 (0.4)	10
JHO-2015-1	2019																16.0 (1.0)	9.9 (0.5)	− 6.1	16.0 (0.2)	9.9 (0.1)	− 6.1
JO-5	2019																0.04 (0.0)	0.04 (0.0)		0.04 (0.0)	0.04 (0.0)	
OS-405	2020																2.0 (0.1)	20.3 (1.1)	18.3	2.0 (0.02)	20.3 (0.2)	18.3
OL-1869-1	2020																9.3 (0.6)	11.3 (0.6)	2	9.3 (0.1)	11.3 (0.1)	2
OL-1861	2020																20.0 (1.3)	6.5 (0.3)	− 13.5	20.0 (0.2)	6.5 (0.1)	− 13.5
OL-1876-2	2020																10.0 (0.7)	7.0 (0.4)	− 3.0	10.0 (0.1)	7.0 (0.08)	− 3
OL-1896	2020																10.0 (0.7)	10.0 (0.5)		10.0 (0.1)	10.0 (0.1)	
OL-1874	2020																1.5 (0.1)	2.0 (0.1)	0.5	1.5 (0.01)	2.0 (0.02)	0.5
**Total**		**676.9**	**570.2**	**− 106.7**	**643.6**	**806.8**	**163.2**	**1,030.7**	**1,540.1**	**509.3**	**2,995.1**	**2,248.3**	**− 746.8**	**1,756.8**	**1,489.8**	**− 267.0**	**1,524.9**	**1,914.4**	**389.6**	**8,628.0**	**8,569.6**	**− 58.4**

*I*, indent (*q*); *P*, production (*q*).

a Net = (Production − Indent), where (−) indicates deficit and (+) indicates surplus BS production.

### 3.4 Institutional BS allocation and production dynamics

**Figure 5:**
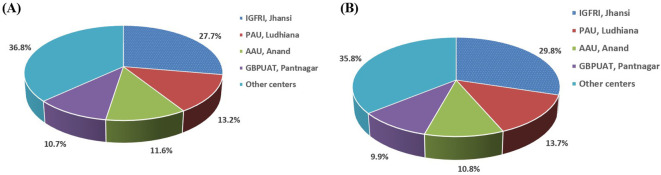
Percentage contribution of the four major AICRP centers to total BS indent **(A)** and production **(B)** in India from 1998–1999 to 2021–2022.

Among the BS-producing institutions, ICAR-IGFRI (Jhansi) was allocated the highest BS indent (23,905 q), followed by PAU (Ludhiana; 1,142.4 q), AAU (Anand; 1,004.9 q), and GBPUAT (Pantnagar; 919.1 q) during 1998–1999 to 2021–2022 (**Figure 5A**). The analysis of production data during the last 24 years reveals that the four centers have contributed more than 64% of the total BS production. ICAR-IGFRI (Jhansi) was the leading center with a production of 2,553 q, followed by PAU (Ludhiana; 1,173.9 q), AAU (Anand; 925.6 q), and GBPUAT (Pantnagar; 845 q) (**Figure 5B**). IGFRI (Jhansi) and PAU (Ludhiana) centers produced more BS than the indent.

Since 1998–1999, a total of 45 fodder oat varieties have entered the seed chain, with 13 institutes participating in the BS program (**Table 4**). Among these, BS production exceeded the indent for 24 varieties over the last 24 years (**Table 3**). However, for the mega variety Kent, BS production faced a deficit of − 478.1 q against an indent of 5,267.8 q, with the maximum deficit reported at GBPUAT (Pantnagar; − 130.0 q), BAIF (Urulikanchan; − 119.2 q), and AAU (Anand, − 79.2 q). IGFRI (Jhansi) produced a surplus BS seed for five varieties (JHO-822, JHO-2009-1, JHO-2010-1, JHO-99-1, and JHO-2012-2) but fell short of the indent for others (**Table 4**). Similarly, PAU (Ludhiana) achieved a surplus BS production for seven varieties (Kent, OL-9, OL-1709, OL-1760, OL-1869-1, OL-1769-1, and OL-1874) over the last 24 years. In addition, SKUAST (Srinagar) was the only center that produced a surplus BS for all indented varieties, whereas other centers experienced shortfalls in one or more varieties. Despite an overall surplus, a varietal mismatch was observed across the centers.

**Table 4:** Fodder oat varietal BS allocation and production status at different institutes in India over the last 24 years (1998–1999 to 2021–2022) (data source: Anonymous, 1999, 2000, 2001, 2002, 2003, 2004, 2005, 2006, 2007, 2008, 2009, 2010, 2011, 2012, 2013, 2014, 2015, 2016, 2017, 2018, 2019, 2020, 2021, 2022).

Variety	Center	Allocation (*q*)	Production (*q*)	Surplus/deficit (*q*)	Surplus/deficit (%)
Kent	IGFRI, Jhansi	1,355.6	1,323.9	− 31.7	− 2.3
	JNKVV, Jabalpur	719.7	665.7	− 54.0	− 7.5
	CCSHAU Hisar	168.0	112.4	− 55.6	− 33.1
	GBPUAT, Pantnagar	230.0	100.0	− 130.0	− 56.5
	SKRAU, Bikaner	220.6	187.5	− 33.1	− 15.0
	PAU, Ludhiana	889.8	897.5	7.7	0.9
	MPKV, Rahuri	188.0	166.7	− 21.3	− 11.3
	AAU, Anand	1,004.9	925.6	− 79.3	− 7.9
	BAIF, Urulikanchan	456.4	337.2	− 119.2	− 26.1
	ANDUAT, Ayodhya	35.0	3.2	− 31.9	− 91.0
	NDRI, Karnal	0.0	70.0	70.0	0.0
JHO-822	IGFRI, Jhansi	319.6	570.6	251.0	78.5
JHO-851	IGFRI, Jhansi	247.0	242.4	− 4.6	− 1.9
JHO-2015-1	IGFRI, Jhansi	16.0	9.9	− 6.1	− 38.1
JHO-2009-1	IGFRI, Jhansi	56.0	63.3	7.3	13.1
JHO-2010-1	IGFRI, Jhansi	20.0	23.4	3.4	17.0
JHO-99-1	IGFRI, Jhansi	21.5	24.5	3.0	13.9
JHO-99-2	IGFRI, Jhansi	250.3	226.3	− 24.0	− 9.6
JHO-2000-4	IGFRI, Jhansi	102.5	63.5	− 39.1	− 38.1
JHO-2012-2	IGFRI, Jhansi	2.0	5.3	3.3	162.5
JO-03-93	JNKVV, Jabalpur	27.5	10.0	− 17.5	− 63.5
JO-04-315	JNKVV, Jabalpur	4.5	1.7	− 2.8	− 62.1
JO-03-91	JNKVV, Jabalpur	18.0	5.0	− 13.0	− 72.2
JO-01	JNKVV, Jabalpur	0.0	46.0	46.0	0.0
JO-5	JNKVV, Jabalpur	0.0	0.0	0.0	0.0
SKO-7	SKUAST, Srinagar	369.0	450.5	81.5	22.1
SKO-96	SKUAST, Srinagar	30.0	34.0	4.0	13.3
SKO-20	SKUAST, Srinagar	146.5	169.5	23.0	15.7
HJ-8	CCS HAU Hisar	190.9	205.8	14.9	7.8
	SKRAU, Bikaner	0.0	5.0	5.0	
OS-6	CCS HAU Hisar	241.2	219.5	− 21.7	− 9.0
	SKRAU, Bikaner	0.0	2.0	2.0	
OS-403	CCS HAU Hisar	84.9	70.5	− 14.4	− 17.0
OS-377	CCS HAU Hisar	41.2	71.0	29.8	72.2
OS-346	CCS HAU Hisar	20.5	35.5	15.0	73.2
OS-7	CCS HAU Hisar	16.0	13.2	− 2.8	− 17.5
HFO-114	CCS HAU Hisar	24.2	18.0	− 6.2	− 25.8
OS-405	CCS HAU Hisar	2.0	20.3	18.3	915.0
UPO-212	GBPUAT, Pantnagar	670.2	721.0	50.8	7.6
UPO-06-1	GBPUAT, Pantnagar	13.3	0.0	− 13.3	− 100.0
UPO-94	GBPUAT, Pantnagar	5.6	24.0	18.4	328.6
OL-9	PAU, Ludhiana	5.3	6.0	0.8	14.3
OL-1709	PAU, Ludhiana	103.6	131.6	28.0	27.0
OL-1760	PAU, Ludhiana	9.0	20.5	11.5	127.8
OL-1802-1	PAU, Ludhiana	39.5	38.5	− 1.0	− 2.5
OL-1869-1	PAU, Ludhiana	9.3	11.3	2.0	21.5
OL-1802	PAU, Ludhiana	20.5	9.0	− 11.5	− 56.1
OL-1769-1	PAU, Ludhiana	24.0	34.0	10.0	41.7
OL-1861	PAU, Ludhiana	20.0	6.5	− 13.5	− 67.5
OL-1876-2	PAU, Ludhiana	10.0	7.0	− 3.0	− 30.0
OL-1896	PAU, Ludhiana	10.0	10.0	0.0	0.0
OL-1874	PAU, Ludhiana	1.5	2.0	0.5	33.3
RO-11-1	MPKV Rahuri	27.0	31.9	4.9	18.2
RO-19	MPKV Rahuri	116.2	109.9	− 6.3	− 5.4
Palampur-1	CSKHPKV Palampur	2.0	2.4	0.4	20.0
NDO-1	NDUAT, Ayodhya	16.0	7.2	− 8.8	− 55.0
NDO-2	NDUAT, Ayodhya	6.0	0.5	− 5.5	− 91.7
**Total**		**8,628.0**	**8,569.6**	**− 58.4**	**− 0.7**

### 3.5 Varietal replacement rate and varietal age

**Figure 6:**
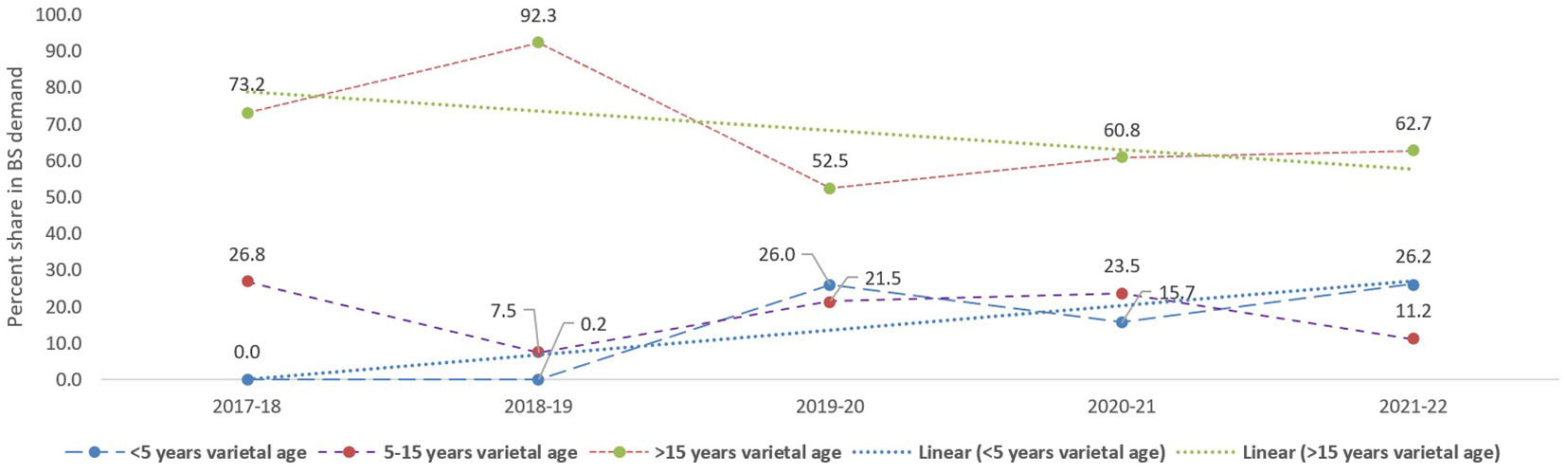
Percentage contribution of fodder oat varieties (< 5, 5–15, and >15 years) to the total BS indent over the last 5 years (2017–2018 to 2021–2022).

VRR is vital in sustaining enhanced crop productivity and provides resilience to biotic and abiotic stresses under changing climatic conditions. Of 45 notified varieties, 34 were in the seed chain, and 1,221.8 q of BS was indented in the last 3 years (2019–2020 to 2021–2022). Of the total BS indented, 23% was from 16 varieties of < 5 years varietal age (**Table 5**). However, the contribution of more than 15-year-old varieties was significantly high (58.7%), emphasizing their popularity among fodder producers. Since 2017–2018, the contribution of new varieties (< 5 years) has significantly increased from 0.2% (2018–2019) to 26.2% (2021–2022), whereas the share of old varieties (> 15 years old) has decreased from 73.2% (2017–2018) to 62.7% (2021–2022) in the last 5 years (**Figure 6**).

**Table 5:** Varietal replacement rate (VRR) in fodder oats in India over the last 3 years (2019–2020 to 2021–2022).

Number of total notified varieties	No. of varieties in the seed chain	Total BS indent (*q*)	Varieties < 5 years old			Varieties < 15 years old			Varieties > 15 years old		
			No.	Indent (*q*)	% share in total indent	No.	Indent (*q*)	% share in total indent	No.	Indent (*q*)	% share in total indent
45	34	1,221.8	16	279.8	22.9	25	504.6	41.3	9	717.2	58.7

### 3.6 Prediction of quality seed production and acreage

The Indian seed system follows a three-generation seed production process: BS to FS to CS. Fodder oat area in cultivation can be predicted based on the availability of quality seed in the seed chain, i.e., BS, FS, and CS. In this study, total BS production was reported at 485.1 q in 22 different notified oat varieties during 2021–2022 (**Figure 2**). The seed multiplication ratio (SMR) is an important parameter for predicting foundation and certified seed. The SMR of fodder oat varieties is reported as 20 (Chauhan et al., 2017). Thus, the total FS and CS production would be 9,702 q and 194,040 q in 2022–2023 and 2023–2024, respectively, if the seed supply chain operates at 100% efficiency and other operations are taken care of very prudently (**Table 6**). The total production of CS is sufficient to sow 0.19 M ha, which constitutes 75% of the total estimated oat cultivation area. However, the informal seed system and truthfully labeled seed availability also influence the actual area under the crop and seed replacement rate. Therefore, their contribution should be considered while estimating the actual area under fodder oats.

**Table 6:** Prediction of foundation and certified seed production in fodder oats based on available breeder seed in India.

Crop	Seed rate (kg/ha)		SMR^a^	Approx. area (M ha)^a^	Seed demand (*q*)			Seed production (*q*)			Estimated area covered (M ha) (2024–2025)
	Fodder production	Seed production			BS (2021–2022)	FS (2022–2023)	CS (2023–2024)	BS (2021–2022)	FS (2022–2023)	CS (2023–2024)	
Oat	100	75	20	0.25	625	12,500	250,000	485.1	9702	194,040	0.19

*SMR*, seed multiplication ratio; *BS*, breeder seed; *FS*, foundation seed; *CS*, certified seed.

a According to Chauhan et al. (2017).

### 3.7 Forecasting of BS production and varietal number

**Figure 7:**
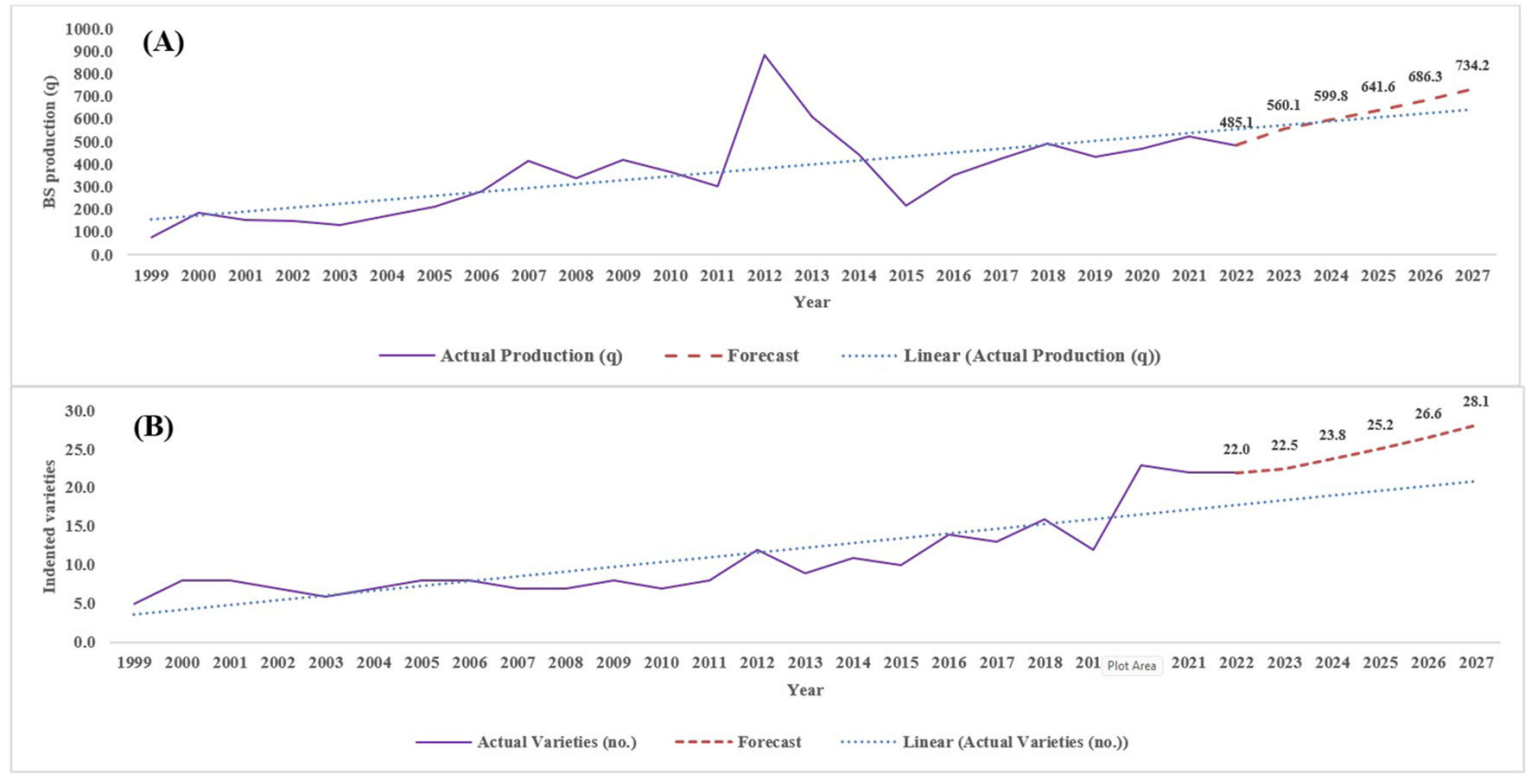
Actual and projected values of fodder oat BS production **(A)** and indented varieties **(B)** in the seed chain.

The parameters of the ARIMA model were estimated and are presented in **Table 7**, followed by the estimation of the model residuals. The (MA1) parameter of the ARIMA model was found to be significant at the 10% level for BS production and at the 5% level for varieties and incidents, with the lowest AIC value. The forecasted values obtained from the ARIMA (0, 0, 1) model are depicted in **Figures 7A, B**, covering the next 5 years (2022–2023 to 2026–2027). The forecasted values of BS production (**Figure 7A**) and indented varieties (**Figure 7B**) indicate an increasing trend. The prediction model estimated a linear increase in BS production, rising from 485.1 q in 2021–2022 to an expected 734.2 q in 2026–2027. Similarly, the number of indented varieties, which was 22 in 2021–2022, is projected to increase to 28 by 2026–2027.

**Table 7:** Estimated (MA1) parameter of the ARIMA model for BS production and indented variety prediction.

Parameters	Estimate	S.E.	*t*-stat	*p*-value
Breeder seed production	− 0.5452	0.0387	− 1.766	0.092
Number of varieties	− 0.6224	0.2712	− 2.29	0.032

Source: authors estimate.

## 4 Discussion

### 4.1 Varietal diversification and narrow genetic base

Varietal diversification supports sustainable crop production and farm profitability by enhancing resilience to biotic and abiotic stresses (Chauhan et al., 2021; Ahmed et al., 2023a, b). However, a narrow genetic base in varietal development can increase the risk of disease and pest outbreaks over time (King and Lively, 2012). In fodder oats, over 75% of varietal development has relied on old varieties (Kent, OS-6, and OS-7) as parents, indicating high parental homogeneity and a limited genetic base. Consequently, fodder oat varieties remain susceptible to leaf blight disease (*Pyrenophora avenae*), particularly under artificial conditions at the seedling stage (Hilli et al., 2023), with their narrow genetic base likely being a key to disease susceptibility. Varietal diversification offers end-users a wider selection of varieties suited to their agroclimatic conditions, farming systems, and specific purposes (Chand et al., 2022). In fodder oats, hybridization followed by selection is the most common breeding approach. Notably, > 70% of varieties have been developed using the pedigree method, followed by pure-line selection and mutation breeding. In India, fodder oat breeding programs have significantly strengthened over the past two decades, leading to the concurrent development of numerous varieties. Moreover, regeneration ability and rapid growth are essential traits for multicut fodder oats to ensure a consistent supply of green fodder during lean periods (Roy et al., 2020; Indu et al., 2023). However, the majority of available varieties are single-cut (28), followed by multicut (13), and dual-purpose (7) types. This highlights the need to reorient breeding strategies toward developing more multicut and dual-purpose fodder oat varieties.

### 4.2 BS production and indent scenario

The increasing trend of notified varieties in the seed chain reflects the intensive efforts of government agencies and the growing demand from stakeholders for different agro-climatic conditions and farming systems. BS production by seed multiplication agencies is directly proportional to BS indent (Chand et al., 2023), with higher indents leading to increased production. The rising BS indent across different phases indicates the importance of oat green fodder among livestock keepers in meeting fodder and nutritional requirements. BS production exceeded the indent during the second, third, and sixth phases, whereas it fell short in the first, fourth, and fifth phases. The BS indent for the variety Kent was remarkably high in 2011–2012, 2012–2013, and 2017–2018. However, the institutes were unable to meet this demand due to insufficient nucleus seed (Chand et al., 2023). Despite these challenges, the concerted and continuous efforts of seed production agencies and other stakeholders have sustained the BS production program within a stable range, ensuring fodder availability over the past 24 years. Additionally, BS production was nearly equal to the indent, with only a 0.68% deficit over nearly two and half decades. This reflects the commendable efforts of BS production agencies in meeting the indent despite the challenges posed by unpredictable weather conditions.

An increased BS indent for a particular variety over the years signifies its dominance over others, as mega varieties tend to be more acclimatized and resilient to biotic and abiotic stresses and/or benefit from sufficient seed availability (Chauhan et al., 2021; Chand et al., 2023). For instance, the variety Kent alone accounts for more than 60% of the BS indent from 1998–1999 to 2021–2022, owing to its multipurpose use (single, multicut, and dual type), high green fodder yield (550–570 q/ha), and tolerance to lodging and seed shattering. However, its demand has significantly declined from 79.4% (phase I) to 34.3% (phase VI), indicating the rapid introduction of new high-yielding varieties into the seed chain. Furthermore, the sustained dominance of older varieties such as Kent, UPO-212, JHO-822, OS-6, JHO-851, and HJ-8 (released before 1998) underscores their popularity among farmers, attributed to their wide adaptability, easy access to quality seed, lodging tolerance, non-seed-shattering nature, and high green fodder yield (Roy et al., 2020). Of 45 oat varieties, BS production was either surplus or equal to BS indent for 25 varieties, whereas it fell short for others. Among the older but widely adopted varieties, BS production exceeded indent for JHO-822, UPO-212, and HJ-8, whereas it fell short for Kent, OS-6, and JHO-851. A total of 13 national and state institutes were involved in oat BS production, with major contributors including ICAR-IGFRI (Jhansi), PAU (Ludhiana), AAU (Anand), and GBPUAT (Pantnagar), collectively accounting for more than 60% of BS production since 1998–1999. However, varietal BS production fluctuates over the years due to genotypic and environmental factors, such as rainfall during the flowering stage and other agronomic conditions (Chand et al., 2023). Optimized agronomic management practices can enhance seed production; for instance, BS production at ICAR-IGFRI (Jhansi) exceeded indent by 6.8%, contributing 29.8% to the total BS production from 1998–1999 to 2021–2022.

### 4.3 Varietal replacement rate and varietal age

The development and deployment of improved varieties contribute to higher economic yields, enhanced nutritional quality, greater tolerance to diseases and pests, and better adaptability to evolving environmental conditions, diverse agro-climatic regions, and cropping systems (Singh et al., 2020; Chand et al., 2023). In fodder oats, the contribution of old varieties (> 15 years) to BS indent has significantly decreased from 92.3% (2018–2019) to 62.7% (2021–2022) over the past 5 years (2017–2018 to 2021–2022). Conversely, the share of varieties developed within the last 5 years has risen from 0.2% (2017–2018) to 26.2% (2021–2022), indicating the increasing adoption of newly developed varieties by end-users. Varietal development and introgression of newly released varieties into the seed chain is a continuous process. However, only a few varieties have dominated over the years in the total BS indent (Singh et al., 2020; Chand et al., 2023). In fodder oats, high VRR (≈ 23%) has been observed in the last 3 years (2019–2020 to 2021–2022) for varieties with < 5 years of varietal age in total BS indent. However, old varieties (> 15 years) dominate and contribute nearly 59% of the total BS indent. Likely, the highest VRR was reported for wheat, followed by mung bean and chickpea in India. For instance, the contribution of recently developed wheat varieties (< 5 years of age) is 45.3% during 3 years (2017–2018 to 2019–2020) (Singh et al., 2020). Recently, moderate (23.67%) and high (43.30%) VRR for the lucerne (Chand et al., 2023) and berseem (Chand et al., 2024) varieties (< 5 years of age) in the last 3 years (2019–2020 to 2021–2022) have been reported. At the government level, policies should focus on encouraging high-yielding varieties, bolstering seed quality control systems, ensuring farmers have easy access to high-quality seed, promoting public–private partnerships for seed storage and distribution, and raising awareness among farmers about the benefits of using certified seed to safeguard their interests and maintain agro-biodiversity.

### 4.4 Area coverage

Crop acreage mainly depends on its economic importance, benefit–cost ratio, available resources, and agro-climatic conditions. Moreover, the availability of quality seed also influences crop acreage, as spurious seeds result in low productivity and poor farm profitability. Quality seeds ensure high productivity while keeping cultivation costs low. To meet the existing 0.25 M ha area under cultivation with a 100% seed replacement rate, India requires 625 q of BS per annum (Chauhan et al., 2017). However, the existing BS availability meets only 77.6% of the commercial seed requirement. The remaining demand is likely fulfilled through truthfully labeled seeds from public and private sectors or informal seed systems, viz., farm-saved seed, farmer-to-farmer seed exchanges, and noncertified seeds from local shops.

### 4.5 Agronomic interventions for enhanced seed yield

Unlike other crops, where grain or its derived products hold economic value, forage crops are typically harvested between the preflowering and 50% flowering stages, yielding a high volume of green forage without compromising nutritional penalty (Malik et al., 2015; Singhal et al., 2022). Fodder crops, including fodder oats, are mainly bred for vigorous vegetative growth. However, high biomass production at an early stage is often negatively associated with seed yield due to lodging. Additionally, in single-cut and taller oat varieties, excessive soil fertility and high nitrogen application can cause lodging at the flowering stage, negatively affecting seed production (Berry et al., 2003). Therefore, the cultivation practices for seed and fodder production differ. Several factors, including the selection of suitable agro-climatic conditions, date of sowing and seed rate, fertilizer application, water and weed management, plant protection, and harvesting methods, play a crucial role. In India, quality seed production is mainly concentrated in Punjab, Haryana, Uttar Pradesh, and Madhya Pradesh, where climatic conditions are favorable for crop growth and development. Previously, May et al. (2004) reported that early seeding and optimal N fertilizer application resulted in higher oat seed yield and test weight. However, taller fodder oat genotypes may exhibit excessive growth when sown early, making them more prone to lodging due to erratic heavy rainfall, high wind velocity, and hailstorms. Regarding N application, a high seed yield has been reported with 80 kg N/ha, while single-cut oat requires 120 kg N/ha to achieve high fodder volume (Joshi et al., 2015). Similarly, sowing in the first fortnight of November is recommended for maximizing forage biomass. In contrast, sowing in the second fortnight of November to the first week of December is recommended for promoting good tillering and optimal vegetative growth while allowing sufficient time for grain formation and filling. This timing leads to higher seed yields and enhances tolerance to adverse climatic conditions, such as high-velocity winds and hailstorms, which typically occur in February and March (Niu et al., 2016). Seed yield is primarily influenced by seed rate and seed quality. For forage production, an oat seed rate of 80–100 kg/ha is recommended, whereas 50–60 kg is suggested for seed production. Additionally, seed size affects the required seed rate, as bold-seeded varieties (e.g., Kent, JHO-822) require a higher seed rate. Fertilizer management is crucial for achieving optimal seed yield. For instance, grain yield has been reported to increase with nitrogen application up to 90 kg N/ha; however, exceeding this level can lead to crop lodging and increased weed infestation, resulting in yield penalties (Bhat et al., 2000). Effective weed management is also essential, as unchecked weeds can significantly reduce seed yield. Depending on the weed population’s nature and persistence, yield losses can range from 20% to 40%. Broadleaf and grassy weeds are predominantly found in oat fields and can be managed using a weeder-cum-mulcher at the 4-week crop stage, followed by the application of 2,4-D at 0.37 kg a.i./ha at the 6-week stage. Additionally, the application of metsulfuron-methyl at 8 g/ha combined with one-hand weeding has been identified as the most effective chemical weed control method (Kumar et al., 2012).

## 5 Conclusion

Oat is an introduced crop in India. Over the past decades, a significant amount of germplasm has been introduced from several gene banks, evaluated, and utilized in breeding programs. Most popular varieties share a common ancestry. There is a need to expand parental heterogeneity by incorporating more germplasm into hybridization programs to enhance yield and stress tolerance. Two wild species, *A. sterilis* and *A. maroccana*, have been successfully used in several institutions to broaden the genetic base. The recent increase in varietal diversification across different oat-growing regions has enhanced seed production. Furthermore, expanding dual-purpose varieties will further improve crop value addition and overall production. The BS indent directly impacts certified seed production and availability.

The availability of high-quality seeds for suitable new oat varieties has increased the varietal replacement rate and reduced the dependency on Kent, the mega oat varieties, by 50% in the last few years. Therefore, indenting old varieties should be discouraged by promoting suitable replacement varieties through demonstrations and government seed programs. A seed-rolling plan can be developed for the next 10 years to meet seed requirements based on predicted values. In addition, the government could provide additional financial assistance to farmers for cultivating newly released varieties, thereby increasing the VRR and promoting their adoption. The production and availability of high-quality fodder seed, including oats, can be improved by implementing varietal-specific seed production technology while considering the differences between commercial utilization products and their seeds.

The country can expand green fodder production and improve access to quality fodder seeds by implementing specific policy changes. Encouraging the private sector, particularly those with extensive marketing networks, can accelerate the adoption of new varieties in crop-growing regions. Nonexclusive business agreements, limited to a few private organizations for multiplying and marketing highly productive new varieties, will simultaneously incentivize research institutes and benefit the farming community. Encouraging dual-purpose fodder crops like oats—where green fodder can initially be used for livestock and later harvested as grain for human consumption—will help reduce competition between food and fodder crops, making it a boon for limited land resources. Future breeding efforts should focus on developing high-fodder-yielding varieties with good seed production, as well as exclusively dual-purpose varieties. Additionally, the development of multicut varieties should remain a priority, as they provide farmers with green, nutritious fodder over an extended period. High vegetative growth and industry-required grain quality should be incorporated as criteria for varietal release. The additional economic benefits will encourage farmers to adopt new varieties more quickly. Special government promotion through appropriate funding for often neglected crops, including fodder seed production by farmer–producer organizations (FPOs) and seed distribution through the multistate cooperative society, Bharatiya Beej Sahakari Samithi Limited (BBSSL)—where the National Dairy Development Board (NDDB) is one of the promoters—will bring a significant improvement in fodder availability in India.

## Data Availability

The original contributions presented in the study are included in the article/**Supplementary Material**. Further inquiries can be directed to the corresponding authors.

## Supplementary material

Supplementary Figure 1
**(A, B)** Akaike Information Criteria (AIC) values for 20 models were used to select the best ARIMA model for breeder seed production **(A)** and indented varieties **(B)**.
