# Supplementary figures and images for: Analyzing trends and future projections in fodder oats (Avena sativa L.) for quality seed production in India
Source: Front Plant Sci. 2025 Apr 7;16:1525422. doi: 10.3389/fpls.2025.1525422 (PMC12009856; doi:10.3389/fpls.2025.1525422)

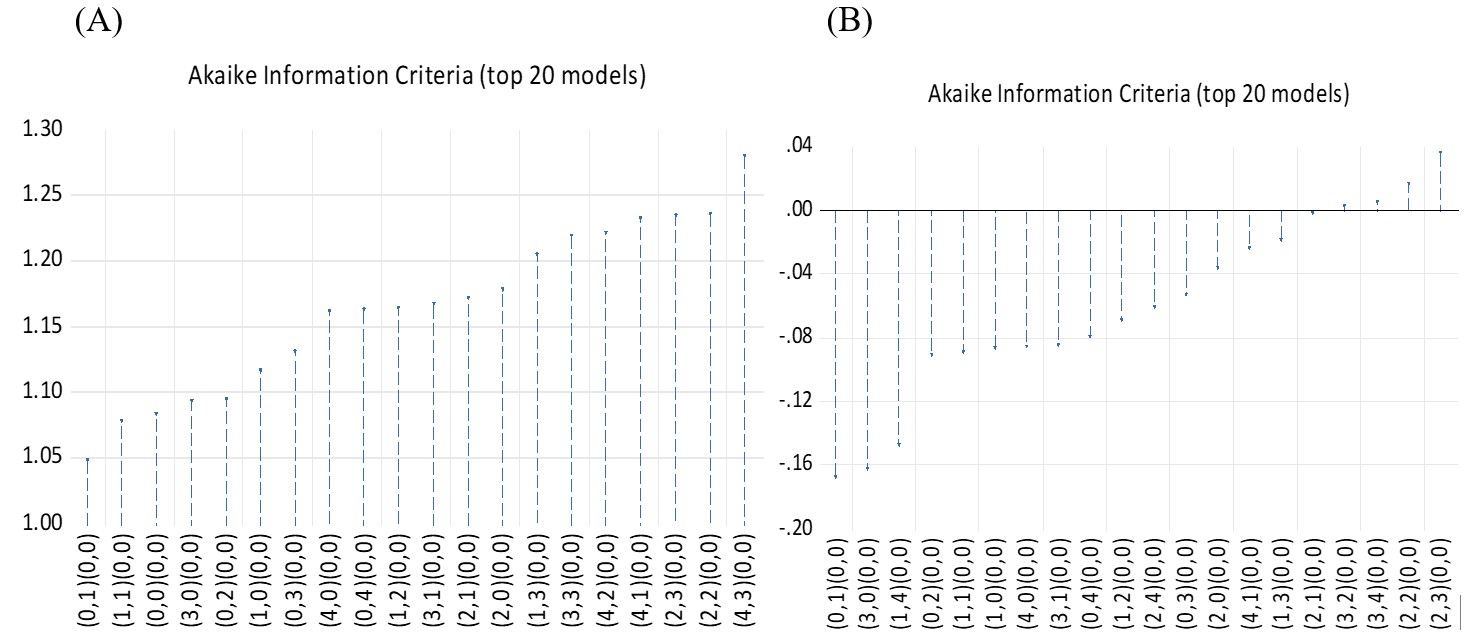

Supplement: Supplementary Figure 1 — (A, B) Akaike Information Criteria (AIC) values for 20 models were used to select the best ARIMA model for breeder seed production (A) and indented varieties (B). [file Image1.jpg]
